# Supplementary material for: In Vitro Activity of Imipenem/Relebactam Alone and in Combination Against Cystic Fibrosis Isolates of Mycobacterium abscessus
Source: Antibiotics (Basel). 2025 May 10;14(5):486. doi: 10.3390/antibiotics14050486 (PMC12108374; doi:10.3390/antibiotics14050486)
Supplement: Supplementary file 1 [file antibiotics-14-00486-s001.zip › antibiotics-3633726-supplementary.pdf]

Supplementary Materials:

Table S1. *M. abscessus* CF clinical isolates identified according to their subspecies classification and morphology.

CF Patient ASID	WGS Identification	CF Morphology
CF00006	MAB	R
CF00013	MAB	R
CF00016	MAB	R
CF00017	MAB	R
CF00023	MAB	R
CF00038	MAB	S
CF00040	MAB	R
CF00041	MAB	S
CF00043	MAB	R
CF00136	MAB	intermediate
CF00258	MAB	intermediate
CF00855	MAB	intermediate
CF01975	MAB	R
CF02033	MAB	R
CF02279	MAB	R
CF02319	MAB	R
CF02486	MAB	S
CF00008	MMAS	R
CF00030	MMAS	S
CF00035	MMAS	R
CF00042	MMAS	S
CF00046	MMAS	intermediate
CF00047	MMAS	S
CF00883	MMAS	R
CF00020	MBOL	S
CF00113	MBOL	intermediate
CF00868	MBOL	intermediate
CF02061	MBOL	R

MAB, *M. abscessus* subsp. *abscessus*; MBOL, *M. abscessus* subsp. *bolletii*; MMAS, *M. abscessus* subsp. *massiliense*; R, rough; S, smooth.

Table S2. Minimum inhibitory concentrations (MICs) of amoxicillin and imipenem/relebactam (IMI/REL), both individually and in combination against the *M. abscessus* CF clinical isolates, along with the corresponding fractional inhibitory concentration (FIC) index values and their synergism.

WGS ID	CF Patient ID	MIC Value (μg/mL)				FIC Index	Interaction
		Amoxicillin Alone	Amoxicillin with IMI/REL	IMI/REL Alone	IMI/REL with Amoxicillin		
M. abscessus	CF00006	2048	256	16	8	**0.625**	Additive
	CF00013	2048	512	2	0.5	**0.500**	Synergistic
	CF00016	2048	512	8	2	**0.500**	Synergistic
	CF00017	1024	64	4	2	**0.563**	Additive
	CF00023	2048	64	4	0.5	**0.156**	Synergistic
	CF00038	2048	16	4	1	**0.258**	Synergistic
	CF00040	2048	16	8	8	**1.008**	Indifferent
	CF00041	2048	256	4	1	**0.375**	Synergistic
	CF00043	2048	256	4	1	**0.375**	Synergistic
	CF00136	2048	512	8	1	**0.375**	Synergistic
	CF00258	2048	256	8	4	**0.625**	Additive
	CF00855	2048	256	4	1	**0.375**	Synergistic
	CF01975	1024	128	16	4	**0.375**	Synergistic
	CF02033	2048	256	8	2	**0.375**	Synergistic
	CF02279	2048	64	4	2	**0.531**	Additive
	CF02319	2048	8	2	2	**1.004**	Indifferent
	CF02486	1024	256	8	4	**0.750**	Additive
M. massiliense	CF00008	2048	8	4	2	**0.504**	Additive
	CF00030	2048	64	8	0.5	**0.094**	Synergistic
	CF00035	2048	2048	8	1	**1.125**	Indifferent
	CF00042	1024	32	16	4	**0.281**	Synergistic
	CF00046	1024	256	16	8	**0.750**	Additive
	CF00047	2048	32	8	2	**0.266**	Synergistic
	CF00883	512	16	8	0.125	**0.047**	Synergistic
M. bolletii	CF00020	1024	512	8	2	**0.750**	Additive
	CF00113	1024	128	8	2	**0.375**	Synergistic
	CF00868	2048	64	8	2	**0.281**	Synergistic
	CF02061	2048	32	4	2	**0.516**	Additive
**MIC_{50}**		2048	128	8	2	—	—
**MIC_{90}**		2048	512	16	4.9	—	—

Table S3. Minimum inhibitory concentrations (MICs) of cefoxitin and imipenem/relebactam (IMI/REL), both individually and in combination against the *M. abscessus* CF clinical isolates, along with the corresponding fractional inhibitory concentration (FIC) index values and their synergism.

WGS ID	CF Patient ID	MIC Value (µg/mL)				FIC Index	Interaction
		Cefoxitin Alone	Cefoxitin with IMI/REL	IMI/REL Alone	IMI/REL with Cefoxitin		
M. abscessus	CF00006	64	4	16	16	**1.063**	Indifferent
	CF00013	128	32	8	2	**0.500**	Synergistic
	CF00016	64	16	8	2	**0.500**	Synergistic
	CF00017	32	8	4	2	**0.750**	Additive
	CF00023	64	16	4	1	**0.500**	Synergistic
	CF00038	32	8	16	2	**0.375**	Synergistic
	CF00040	128	8	32	32	**1.063**	Indifferent
	CF00041	32	8	4	1	**0.500**	Synergistic
	CF00043	64	8	4	1	**0.375**	Synergistic
	CF00136	32	8	4	1	**0.500**	Synergistic
	CF00258	64	8	8	1	**0.250**	Synergistic
	CF00855	128	8	64	32	**0.563**	Additive
	CF01975	32	4	64	8	**0.250**	Synergistic
	CF02033	64	8	32	4	**0.250**	Synergistic
	CF02279	64	8	4	1	**0.375**	Synergistic
	CF02319	64	2	8	2	**0.281**	Synergistic
	CF02486	32	4	8	2	**0.375**	Synergistic
M. massiliense	CF00008	64	4	16	8	**0.563**	Additive
	CF00030	32	4	16	4	**0.375**	Synergistic
	CF00035	32	4	8	8	**1.125**	Indifferent
	CF00042	16	4	16	16	**1.250**	Indifferent
	CF00046	64	4	16	8	**0.563**	Additive
	CF00047	32	4	4	1	**0.375**	Synergistic
	CF00883	16	4	8	4	**0.750**	Additive
M. bolletii	CF00020	32	4	16	4	**0.375**	Synergistic
	CF00113	64	8	4	1	**0.375**	Synergistic
	CF00868	32	8	8	2	**0.500**	Synergistic
	CF02061	32	8	4	1	**0.500**	Synergistic
**MIC_{50}** 2048		45.3	8	8	2	—	—
**MIC_{90}** 2048		78.8	9.8	32	16	—	—

Table S4. Minimum inhibitory concentrations (MICs) of cefdinir and imipenem/relebactam (IMI/REL), both individually and in combination against the *M. abscessus* CF clinical isolates, along with the corresponding fractional inhibitory concentration (FIC) index values and their synergism.

WGS ID	CF Patient ID	MIC Value (μg/mL)				FIC Index	Interaction
		Cefdinir Alone	Cefdinir with IMI/REL	IMI/REL Alone	IMI/REL with Cefdinir		
M. abscessus	CF00006	256	128	16	0.125	**0.508**	Additive
	CF00013	256	32	4	1	**0.375**	Synergistic
	CF00016	128	16	4	1	**0.375**	Synergistic
	CF00017	256	8	2	0.5	**0.281**	Synergistic
	CF00023	256	32	4	1	**0.375**	Synergistic
	CF00038	256	64	4	1	**0.500**	Synergistic
	CF00040	256	128	8	1	**0.625**	Additive
	CF00041	256	32	8	1	**0.250**	Synergistic
	CF00043	128	8	4	0.5	**0.188**	Synergistic
	CF00136	16	16	16	0.5	**1.031**	Indifferent
	CF00258	256	128	16	2	**0.625**	Additive
	CF00855	64	16	4	0.5	**0.375**	Synergistic
	CF01975	256	32	8	0.125	**0.141**	Synergistic
	CF02033	256	64	4	1	**0.500**	Synergistic
	CF02279	512	32	2	0.25	**0.188**	Synergistic
	CF02319	128	8	4	1	**0.313**	Synergistic
	CF02486	256	16	4	1	**0.313**	Synergistic
M. massiliense	CF00008	256	64	8	1	**0.375**	Synergistic
	CF00030	128	8	4	1	**0.313**	Synergistic
	CF00035	128	16	8	1	**0.250**	Synergistic
	CF00042	16	16	8	0.125	**1.016**	Indifferent
	CF00046	256	64	8	0.5	**0.313**	Synergistic
	CF00047	128	32	4	1	**0.500**	Synergistic
	CF00883	8	2	4	2	**0.750**	Additive
M. bolletii	CF00020	64	8	4	1	**0.375**	Synergistic
	CF00113	64	8	4	2	**0.625**	Additive
	CF00868	128	32	8	1	**0.375**	Synergistic
	CF02061	64	16	4	1	**0.500**	Synergistic
**MIC_{50}**		181	22.6	4	1	—	—
**MIC_{90}**		256	78.8	9.8	1.2	—	—

Table S5. Minimum inhibitory concentrations (MICs) of cefuroxime and imipenem/relebactam (IMI/REL), both individually and in combination against the *M. abscessus* CF clinical isolates, along with the corresponding fractional inhibitory concentration (FIC) index values and their synergism.

WGS ID	CF Patient ID	MIC Value (µg/mL)				FIC Index	Interaction
		Cefuroxime Alone	Cefuroxime with IMI/REL	IMI/REL Alone	IMI/REL with Cefuroxime		
M. abscessus	CF00006	512	128	8	0.5	**0.313**	Synergistic
	CF00013	512	128	4	0.125	**0.281**	Synergistic
	CF00016	256	64	16	0.5	**0.281**	Synergistic
	CF00017	512	32	4	0.5	**0.188**	Synergistic
	CF00023	512	64	4	0.5	**0.250**	Synergistic
	CF00038	64	16	4	0.125	**0.281**	Synergistic
	CF00040	512	64	8	0.5	**0.188**	Synergistic
	CF00041	256	64	4	0.25	**0.313**	Synergistic
	CF00043	256	16	4	0.125	**0.094**	Synergistic
	CF00136	128	16	16	0.125	**0.133**	Synergistic
	CF00258	256	64	8	1	**0.375**	Synergistic
	CF00855	512	128	8	0.5	**0.313**	Synergistic
	CF01975	256	128	16	1	**0.563**	Additive
	CF02033	1024	256	4	0.5	**0.375**	Synergistic
	CF02279	512	32	2	0.25	**0.188**	Synergistic
	CF02319	512	64	4	0.5	**0.250**	Synergistic
	CF02486	512	32	2	0.125	**0.125**	Synergistic
M. massiliense	CF00008	1024	128	4	0.125	**0.156**	Synergistic
	CF00030	1024	64	4	0.125	**0.094**	Synergistic
	CF00035	512	64	4	0.25	**0.188**	Synergistic
	CF00042	64	32	16	0.25	**0.516**	Additive
	CF00046	256	32	4	0.25	**0.188**	Synergistic
	CF00047	256	64	4	0.5	**0.375**	Synergistic
	CF00883	1024	512	16	2	**0.625**	Additive
M. bolletii	CF00020	512	64	8	0.25	**0.156**	Synergistic
	CF00113	2048	256	8	2	**0.375**	Synergistic
	CF00868	128	16	4	0.125	**0.156**	Synergistic
	CF02061	512	64	4	0.5	**0.250**	Synergistic
**MIC_{50}**		512	64	4	0.4	—	—
**MIC_{90}**		1024	157.6	16	1	—	—

Table S6. Minimum inhibitory concentrations (MICs) of moxifloxacin and imipenem/relebactam (IMI/REL), both individually and in combination against the *M. abscessus* CF clinical isolates, along with the corresponding fractional inhibitory concentration (FIC) index values and their synergism.

WGS ID	CF Patient ID	MIC Value (µg/mL)				FIC Index	Interaction
		Moxifloxacin Alone	Moxifloxacin with IMI/REL	IMI/REL Alone	IMI/REL with Moxifloxacin		
M. abscessus	CF00006	8	4	4	2	**1.000**	Additive
	CF00013	16	8	8	2	**0.750**	Additive
	CF00016	32	16	16	2	**0.625**	Additive
	CF00017	16	4	4	2	**0.750**	Additive
	CF00023	16	4	8	2	**0.500**	Synergistic
	CF00038	16	4	4	2	**0.750**	Additive
	CF00040	16	4	4	1	**0.500**	Synergistic
	CF00041	16	4	4	2	**0.750**	Additive
	CF00043	8	2	4	1	**0.500**	Synergistic
	CF00136	8	2	8	2	**0.500**	Synergistic
	CF00258	16	8	4	2	**1.000**	Additive
	CF00855	8	4	8	2	**0.750**	Additive
	CF01975	8	4	4	1	**0.750**	Additive
	CF02033	16	4	8	2	**0.500**	Synergistic
	CF02279	4	2	4	0.5	**0.625**	Additive
	CF02319	16	8	4	2	**1.000**	Additive
	CF02486	16	4	8	2	**0.500**	Synergistic
M. massiliense	CF00008	16	4	8	2	**0.500**	Synergistic
	CF00030	16	4	8	2	**0.500**	Synergistic
	CF00035	8	4	8	4	**1.000**	Additive
	CF00042	16	4	4	1	**0.500**	Synergistic
	CF00046	8	4	8	2	**0.750**	Additive
	CF00047	8	4	4	1	**0.750**	Additive
	CF00883	4	2	4	2	**1.000**	Additive
M. bolletii	CF00020	8	4	8	2	**0.750**	Additive
	CF00113	8	4	4	2	**1.000**	Additive
	CF00868	16	8	8	2	**0.750**	Additive
	CF02061	16	4	8	2	**0.500**	Synergistic
**MIC_{50}**		16	4	5.7	2	—	—
**MIC_{90}**		16	8	8	2	—	—

Table S7. Minimum inhibitory concentrations (MICs) of azithromycin and imipenem/relebactam (IMI/REL), both individually and in combination against the *M. abscessus* CF clinical isolates, along with the corresponding fractional inhibitory concentration (FIC) index values and their synergism.

WGS ID	CF Patient ID	MIC Value (μg/mL)				FIC Index	Interaction
		Azithromycin Alone	Azithromycin with IMI/REL	IMI/REL Alone	IMI/REL with Azithromycin		
M. abscessus	CF00006	4	1	16	2	**0.375**	Synergistic
	CF00013	2	1	8	1	**0.625**	Additive
	CF00016	16	8	16	4	**0.750**	Additive
	CF00017	2	1	16	2	**0.625**	Additive
	CF00023	16	4	16	4	**0.500**	Synergistic
	CF00038	2	0.5	8	1	**0.375**	Synergistic
	CF00040	8	2	8	1	**0.375**	Synergistic
	CF00041	4	2	4	0.5	**0.625**	Additive
	CF00043	8	4	4	1	**0.750**	Additive
	CF00136	4	2	4	1	**0.750**	Additive
	CF00258	16	4	16	4	**0.500**	Synergistic
	CF00855	16	4	8	2	**0.500**	Synergistic
	CF01975	16	4	16	4	**0.500**	Synergistic
	CF02033	16	8	16	4	**0.750**	Additive
	CF02279	32	16	8	2	**0.750**	Additive
	CF02319	8	2	8	4	**0.750**	Additive
	CF02486	4	2	8	2	**0.750**	Additive
M. massiliense	CF00008	4	1	16	0.5	**0.281**	Synergistic
	CF00030	2	0.5	8	1	**0.375**	Synergistic
	CF00035	2	0.5	8	1	**0.375**	Synergistic
	CF00042	4	1	8	2	**0.500**	Synergistic
	CF00046	2	1	16	4	**0.750**	Additive
	CF00047	4	2	4	1	**0.750**	Additive
	CF00883	32	16	4	2	**1.000**	Additive
M. bolletii	CF00020	32	16	16	8	**1.000**	Additive
	CF00113	32	4	8	4	**0.625**	Additive
	CF00868	16	8	8	4	**1.000**	Additive
	CF02061	8	2	8	2	**0.500**	Synergistic
**MIC_{50}**		8	2	8	2	—	—
**MIC_{90}**		32	9.8	16	4	—	—

Table S8. Minimum inhibitory concentrations (MICs) of rifabutin and imipenem/relebactam (IMI/REL), both individually and in combination against the *M. abscessus* CF clinical isolates, along with the corresponding fractional inhibitory concentration (FIC) index values and their synergism.

WGS ID	CF Patient ID	MIC Value (µg/mL)				FIC Index	Interaction
		Rifabutin Alone	Rifabutin with IMI/REL	IMI/REL Alone	IMI/REL with Rifabutin		
M. abscessus	CF00006	16	8	16	2	**0.625**	Additive
	CF00013	4	2	4	0.5	**0.625**	Additive
	CF00016	16	16	16	0.125	**1.008**	Indifferent
	CF00017	4	0.5	4	2	**0.625**	Additive
	CF00023	16	8	4	1	**0.750**	Additive
	CF00038	16	4	4	1	**0.500**	Synergistic
	CF00040	16	2	4	2	**0.625**	Additive
	CF00041	8	1	4	2	**0.625**	Additive
	CF00043	16	4	4	1	**0.500**	Synergistic
	CF00136	8	2	16	0.125	**0.258**	Synergistic
	CF00258	16	4	16	2	**0.375**	Synergistic
	CF00855	4	1	4	1	**0.500**	Synergistic
	CF01975	2	1	8	0.5	**0.563**	Additive
	CF02033	8	4	4	0.25	**0.563**	Additive
	CF02279	8	4	2	1	**1.000**	Additive
	CF02319	8	2	4	1	**0.500**	Synergistic
	CF02486	16	1	4	2	**0.563**	Additive
M. massiliense	CF00008	16	4	4	2	**0.750**	Additive
	CF00030	16	4	8	2	**0.500**	Synergistic
	CF00035	4	1	8	2	**0.500**	Synergistic
	CF00042	8	2	4	1	**0.500**	Synergistic
	CF00046	16	1	4	2	**0.563**	Additive
	CF00047	8	2	8	2	**0.500**	Synergistic
	CF00883	2	0.5	4	1	**0.500**	Synergistic
M. bolletii	CF00020	2	0.5	8	1	**0.375**	Synergistic
	CF00113	16	4	4	1	**0.500**	Synergistic
	CF00868	8	4	4	0.5	**0.625**	Additive
	CF02061	8	4	4	0.5	**0.625**	Additive
**MIC_{50}**		8	2	4	1	—	—
**MIC_{90}**		16	4.9	16	2	—	—

Table S9. Minimum inhibitory concentrations (MICs) of clofazimine and imipenem/relebactam (IMI/REL), both individually and in combination against the *M. abscessus* CF clinical isolates, along with the corresponding fractional inhibitory concentration (FIC) index values and their synergism.

WGS ID	CF Patient ID	MIC Value (µg/mL)				FIC Index	Interaction
		Clofazimine Alone	Clofazimine with IMI/REL	IMI/REL Alone	IMI/REL with Clofazimine		
M. abscessus	CF00006	0.5	0.5	4	0.25	**1.063**	Indifferent
	CF00013	0.5	0.25	2	0.25	**0.625**	Additive
	CF00016	0.5	0.5	8	0.25	**1.031**	Indifferent
	CF00017	0.5	0.5	4	0.25	**1.063**	Indifferent
	CF00023	2	1	8	4	**1.000**	Additive
	CF00038	1	0.25	8	4	**0.750**	Additive
	CF00040	2	0.5	8	4	**0.750**	Additive
	CF00041	1	0.25	8	0.25	**0.281**	Synergistic
	CF00043	0.5	0.25	4	2	**1.000**	Additive
	CF00136	2	1	4	2	**1.000**	Additive
	CF00258	2	1	8	1	**0.625**	Additive
	CF00855	1	0.25	8	2	**0.500**	Synergistic
	CF01975	0.5	0.25	16	2	**0.625**	Additive
	CF02033	1	0.25	4	1	**0.500**	Synergistic
	CF02279	2	0.5	8	4	**0.750**	Additive
	CF02319	1	0.5	16	4	**0.750**	Additive
	CF02486	1	0.25	4	1	**0.500**	Synergistic
M. massiliense	CF00008	0.5	0.25	4	2	**1.000**	Additive
	CF00030	2	1	8	1	**0.625**	Additive
	CF00035	1	0.5	16	4	**0.750**	Additive
	CF00042	2	1	8	8	**1.500**	Indifferent
	CF00046	2	1	16	8	**1.000**	Additive
	CF00047	2	1	8	1	**0.625**	Additive
	CF00883	1	0.5	4	1	**0.750**	Additive
M. bolletii	CF00020	1	0.5	16	4	**0.750**	Additive
	CF00113	1	0.5	8	4	**1.000**	Additive
	CF00868	1	0.5	16	4	**0.750**	Additive
	CF02061	1	0.5	8	2	**0.750**	Additive
**MIC_{50}**		1	0.5	8	2	—	—
**MIC_{90}**		2	1	16	4	—	—

Table S10. Minimum inhibitory concentrations (MICs) of minocycline and imipenem/relebactam (IMI/REL), both individually and in combination against the *M. abscessus* CF clinical isolates, along with the corresponding fractional inhibitory concentration (FIC) index values and their synergism.

WGS ID	CF Patient ID	MIC Value (µg/mL)				FIC Index	Interaction
		Minocycline Alone	Minocycline with IMI/REL	IMI/REL Alone	IMI/REL with Minocycline		
M. abscessus	CF00006	256	128	4	2	**1.000**	Additive
	CF00013	256	128	4	1	**0.750**	Additive
	CF00016	512	256	32	8	**0.750**	Additive
	CF00017	256	128	2	2	**1.500**	Indifferent
	CF00023	512	64	4	2	**0.625**	Additive
	CF00038	256	64	4	1	**0.500**	Synergistic
	CF00040	256	128	8	4	**1.000**	Additive
	CF00041	512	128	2	2	**1.250**	Indifferent
	CF00043	256	128	2	2	**1.500**	Indifferent
	CF00136	256	64	2	1	**0.750**	Additive
	CF00258	256	128	8	2	**0.750**	Additive
	CF00855	256	128	8	2	**0.750**	Additive
	CF01975	256	64	8	2	**0.500**	Synergistic
	CF02033	256	128	4	2	**1.000**	Additive
	CF02279	256	128	2	1	**1.000**	Additive
	CF02319	256	64	2	1	**0.750**	Additive
	CF02486	1024	128	8	4	**0.625**	Additive
M. massiliense	CF00008	256	64	8	2	**0.500**	Synergistic
	CF00030	256	16	4	2	**0.563**	Additive
	CF00035	256	32	4	0.5	**0.250**	Synergistic
	CF00042	16	16	4	0.25	**1.063**	Indifferent
	CF00046	512	128	4	1	**0.500**	Synergistic
	CF00047	256	32	2	2	**1.125**	Indifferent
	CF00883	8	8	2	0.125	**1.063**	Indifferent
M. bolletii	CF00020	256	256	4	2	**1.500**	Indifferent
	CF00113	256	64	4	1	**0.500**	Synergistic
	CF00868	8	8	4	0.125	**1.031**	Indifferent
	CF02061	256	64	4	2	**0.750**	Additive
**MIC_{50}**		256	90.5	4	2	—	—
**MIC_{90}**		512	128	8	2.5	—	—

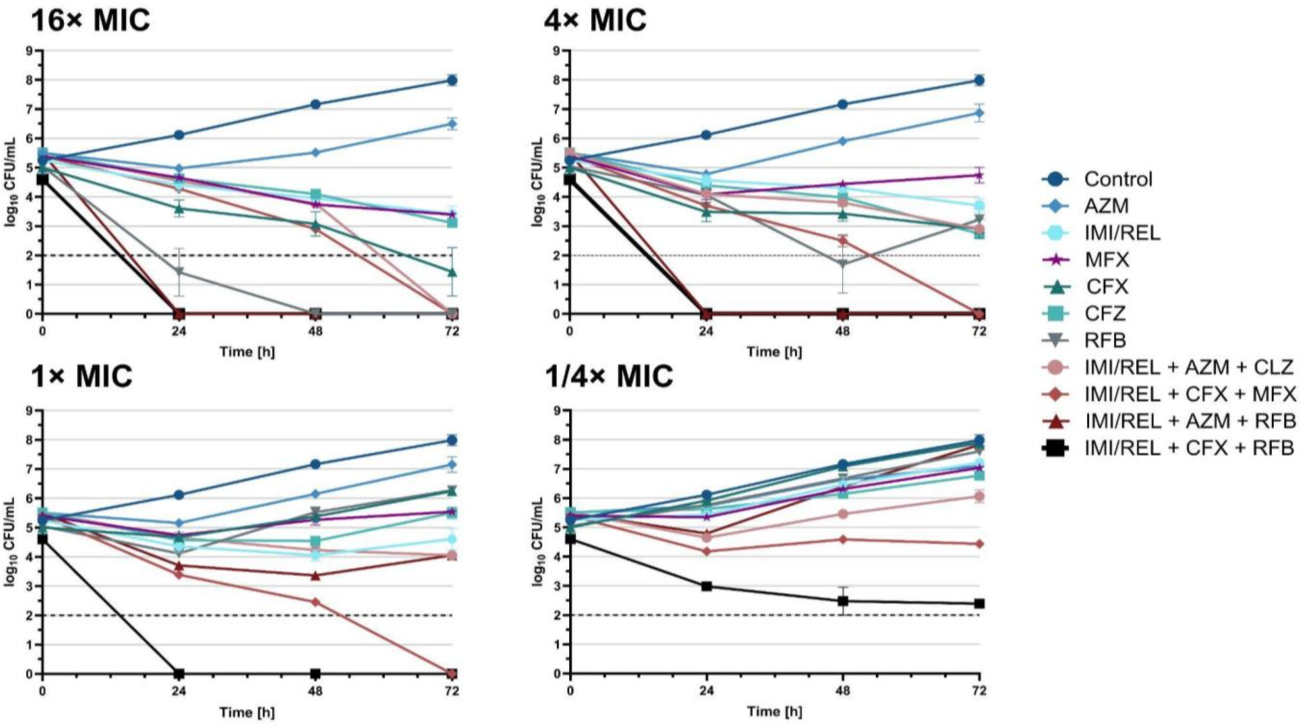

Figure S1. Bacterial load ($\log_{10}$ CFU/ml) of *M. abscessus* ATCC 19977 over 72 hours with single-agent therapies and three-drug combination therapies with imipenem/relebactam (IMI/REL) at 16× MIC, 4× MIC, 1× MIC, and 1/4× MIC. Data are presented as mean with standard errors of the mean. The horizontal dashed line marks the lower limit of detection. MIC = minimum inhibitory concentration, AZM = azithromycin, MFX = moxifloxacin, CFX = cefoxitin, CFZ = clofazimine, RFB = rifabutin

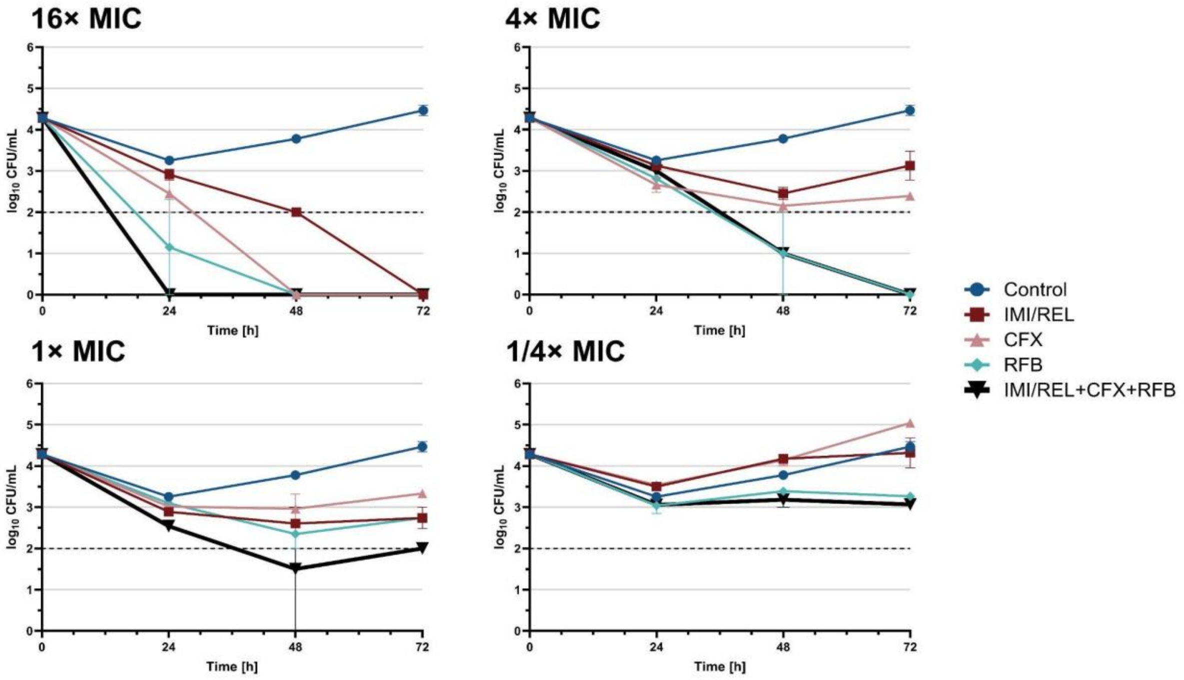

Figure S2. Bacterial load ($\log_{10}$ CFU/ml) of *M. abscessus* CF clinical isolate 13 over 72 hours with imipenem/relebactam (IMI/REL), cefoxitin, rifabutin, and their combination at 16× MIC, 4× MIC, 1× MIC, and 1/4× MIC. Data are presented as mean with standard errors of the mean. The horizontal dashed line marks the lower limit of detection. MIC = minimum inhibitory concentration, CFX = cefoxitin, RFB = rifabutin.

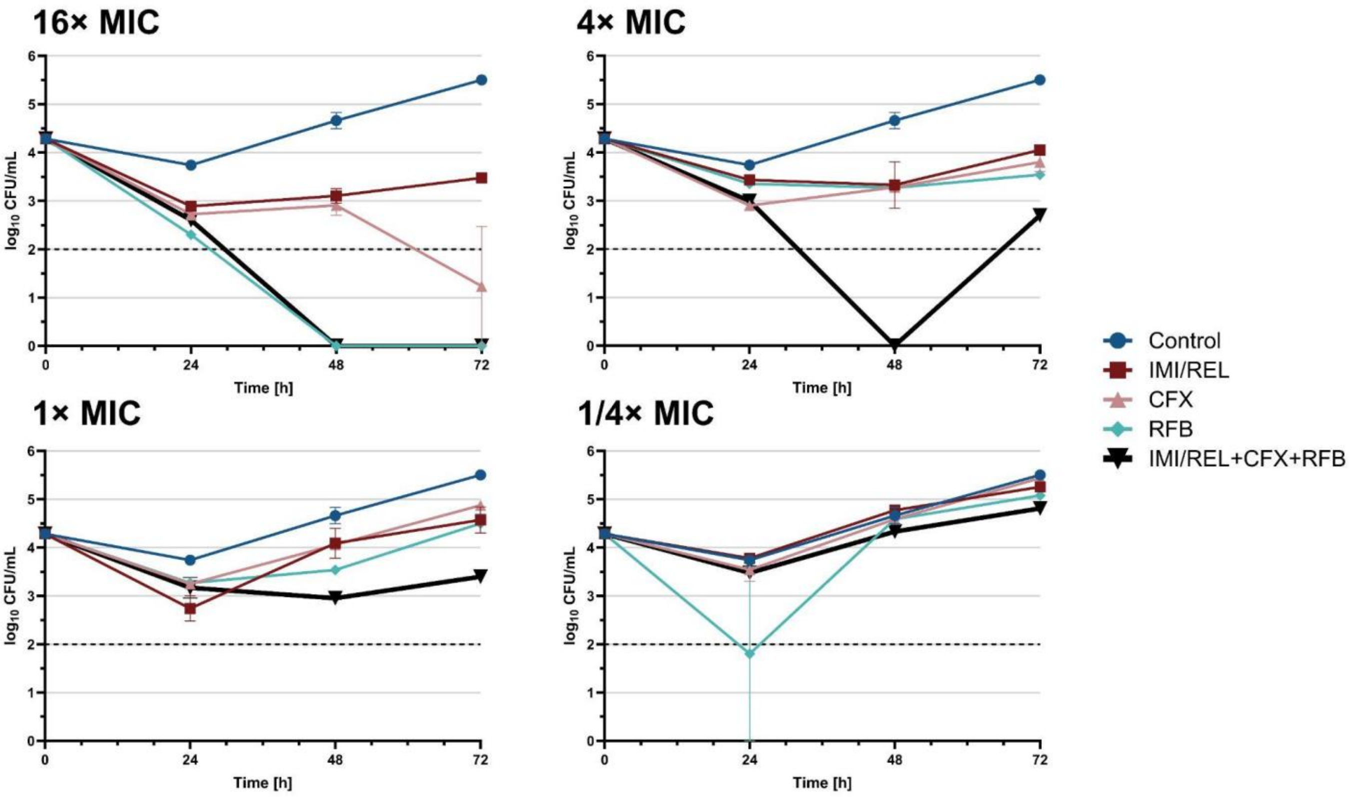

Figure S3. Bacterial load (log_{10} CFU/ml) of *M. abscessus* CF clinical isolate 258 over 72 hours with imipenem/relebactam (IMI/REL), cefoxitin, rifabutin, and their combination at 16× MIC, 4× MIC, 1× MIC, and 1/4× MIC. Data are presented as mean with standard errors of the mean. The horizontal dashed line marks the lower limit of detection. MIC = minimum inhibitory concentration, CFX = cefoxitin, RFB = rifabutin.

Table S11. Minimum inhibitory concentrations (MICs) of imipenem and imipenem/relebactam (IMI/REL) against the *M. abscessus* CF clinical isolates.

WGS ID	CF Patient ID	MIC Value (μg/mL)	
		Imipenem/Relebactam [a]	Imipenem
M. abscessus	ATCC 19977	4	8
	CF00006	16	8
	CF00013	4	8
	CF00016	16	8
	CF00017	4	8
	CF00023	4	8
	CF00038	4	8
	CF00040	8	8
	CF00041	4	8
	CF00043	4	8
	CF00136	8	16
	CF00258	8	8
	CF00855	8	4
	CF01975	16	16
	CF02033	4	8
	CF02279	4	16
	CF02319	4	8
	CF02486	8	8
M. massiliense	CF00008	8	16
	CF00030	8	8
	CF00035	8	8
	CF00042	8	8
	CF00046	8	8
	CF00047	4	8
	CF00883	4	4
M. bolletii	CF00020	8	16
	CF00113	4	4
	CF00868	8	8
	CF02061	4	4
**MIC_{50}**		8	8
**MIC_{90}**		9.2	16

[a] Represents the median MIC value for each strain across all susceptibility assays.
